# Reconstructing Hyperspectral Images from RGB Images by Multi-Scale Spectral–Spatial Sequence Learning

**DOI:** 10.3390/e27090959

**Published:** 2025-09-15

**Authors:** Wenjing Chen, Lang Liu, Rong Gao

**Affiliations:** 1 Hubei Provincial Key Laboratory of Green Intelligent Computing Power Network, Hubei University of Technology, Wuhan 430068, China; chenwenjing@hbut.edu.cn (W.C.); 102311298@hbut.edu.cn (L.L.); 2 School of Computer Science, Hubei University of Technology, Wuhan 430068, China

**Keywords:** Mamba, hyperspectral image, spectral super-resolution, sequence learning

## Abstract

With rapid advancements in transformers, the reconstruction of hyperspectral images from RGB images, also known as spectral super-resolution (SSR), has made significant breakthroughs. However, existing transformer-based methods often struggle to balance computational efficiency with long-range receptive fields. Recently, Mamba has demonstrated linear complexity in modeling long-range dependencies and shown broad applicability in vision tasks. This paper proposes a multi-scale spectral–spatial sequence learning method, named MSS-Mamba, for reconstructing hyperspectral images from RGB images. First, we introduce a continuous spectral–spatial scan (CS3) mechanism to improve cross-dimensional feature extraction of the foundational Mamba model. Second, we propose a sequence tokenization strategy that generates multi-scale-aware sequences to overcome Mamba’s limitations in hierarchically learning multi-scale information. Specifically, we design the multi-scale information fusion (MIF) module, which tokenizes input sequences before feeding them into Mamba. The MIF employs a dual-branch architecture to process global and local information separately, dynamically fusing features through an adaptive router that generates weighting coefficients. This produces feature maps that contain both global contextual information and local details, ultimately reconstructing a high-fidelity hyperspectral image. Experimental results on the ARAD_1k, CAVE and grss_dfc_2018 dataset demonstrate the performance of MSS-Mamba.

## 1. Introduction

Hyperspectral imaging technology is capable of capturing the distribution of multiple continuous spectral bands that can reflect rich physical and chemical information [1], which is different from the traditional RGB imaging mode. Therefore, hyperspectral images (HSIs) have shown significant progress in many fields such as large-area ground object classification [2] and urban construction [3]. Due to the limitations of current imaging technology, HSI still faces challenges such as high time cost of imaging process [4].

Reconstructing hyperspectral images from RGB images, also known as the spectral super-resolution (SSR) technology, provides a low-cost solution to the aforementioned challenge. The goal of SSR is to enhance the spectral resolution of RGB images to match the fineness of HSI. However, recovering multiple channels from the three RGB channels is an ill-posed problem [5]. Based on the theory of sparse coding, early works primarily focuses on extracting prior knowledge from a large number of HSI training samples. This prior knowledge is utilized to generate complete sparse hyperspectral dictionaries [6,7,8], which are then used to reconstruct HSI. However, manual priors are unable to handle tasks in complex environments.

Recently, convolutional neural networks (CNNs) serve as the core framework for most deep learning-based methods. These methods achieve the reconstruction of HSI by learning the nonlinear mapping relationship between RGB images or multispectral images and HSI. However, to ensure computational efficiency, the convolutional kernels in CNNs are usually set to be small. This limits the receptive field of the CNN, thereby affecting its ability to capture global information from the image [9,10,11] and imposing certain constraints on the performance improvement of CNN-based SSR methods.

Recent studies have also pointed out that activating more pixels usually leads to better recovery results [12]. Due to larger receptive fields, transformer-based methods [13,14] typically outperform CNN-based methods. Although the self-attention mechanism offers many advantages, there is an inherent trade-off between global receptive field and computational efficiency. The quadratic computational complexity of the standard Transformer [15,16] is often infeasible for SSR tasks. Although some efficient attention mechanisms, such as shifted window attention [17], can reduce computational costs to a certain extent, they may sacrifice the global receptive field. This indicates that finding a balance between global effectiveness and efficient computation remains a challenge [18].

Recently, the proposal of Mamba [19] has provided new possibilities for balancing global receptive field and computational efficiency. Mamba models long-distance dependencies through the discretized state space equations, and its structured re-parameterization method further reduces computational complexity. This linear complexity characteristic makes it significantly better than Transformers when dealing with long sequences. However, the standard Mamba algorithm was originally designed for sequence data processing and is not suitable for direct application to the SSR task [20]. As a result, many researchers have explored various scanning methods, such as BiDirectional Scan [20] and Cross-Scan [21], to arrange multi-dimensional data into 1D sequences in a specific order. Nevertheless, most current scanning methods only focus on scanning in the spatial domain and neglect the long-range dependencies in the spectral domain. In the field of SSR, the exploration of the spatial–spectral dependency of images using Mamba remains an issue. On the other hand, Transformer [22] typically employs hierarchical downsampling operations to achieve multi-scale feature learning. However, repeated downsampling operations inevitably lead to progressive degradation of spatial details and confusion of spectral and spatial information.

In this paper, we propose a multi-scale spectral–spatial sequence learning Mamba (MSS-Mamba) for SSR. (1) To fully explore the spectral–spatial correlations in images, we integrate the spectral dimension into the sequence scanning process and propose the Continuous Spectral–Spatial Scan (CS3). CS3 rearranges the row, column and band dimensions by first adopting a channel-first scanning strategy to construct sequences enriched with channel features, followed by row scanning to iteratively embed spatial information from different pixels into the sequences, forming channel-row composite sequences. Subsequently, column scanning is applied to vertically connect channel-row sequences. Additionally, by swapping the execution order of row and column scanning, the channel-column sequences of each row are horizontally concatenated to enhance directional diversity learning. (2) Inspired by sequence modeling [23], we design a multi-scale information fusion block (MIF). Following CS3, an additional sequence tokenization step is incorporated, employing sliding window slicing with varying strides and patch sizes to generate sequences containing multi-scale information, where large patches prioritize spatial dimension scanning to learn global spatial relationships, while small patches focus on channel scanning to extract local spectral features. By setting patch sizes larger than or equal to the channel number of feature maps, each generated sequence retains complete spectral information and partial spatial context, thereby strengthening spectral–spatial synergy. The multi-scale sequences are then fed into the Mamba model for feature learning, and their outputs are fused through a routing weight generation module to reconstruct high-quality images. Our method operates directly on the original feature maps without degrading image sizes, while fully leveraging Mamba’s long-sequence modeling capability. Unlike existing Mamba-based vision methods relying on repeated downsampling [21,24,25], our approach addresses the inherent limitations of Mamba in image serialization. The contributions of this paper are as follows:

- We propose a novel MSS-Mamba for SSR tasks, achieving dynamic fusion of spectral–spatial features through multi-scale spectral–spatial sequence joint modeling.
- We design a CS3 mechanism to construct directionally complementary composite sequences, enhancing long-sequence modeling capability while preserving local spatial continuity.
- We conduct experiments on three HSI datasets, and the results demonstrate that the proposed MSS-Mamba outperforms compared methods.

The rest is organized as follows. Section 2 reviews related methods. Section 3 introduces the proposed MSS-Mamba. Section 4 reports the experiments. Finally, Section 5 gives the conclusion.

## 2. Related Works

### 2.1. SSR

The SSR methods can be categorized into traditional methods and deep learning-based approaches. Traditional methods address the spectral reconstruction problem by mining properties of high-dimensional data (e.g., correlations and redundancies) and applying handcrafted priors or assumptions as constraints [26]. For instance, Arad et al. [27] leveraged hyperspectral priors to create hyperspectral features while developing sparse dictionaries of corresponding RGB projections for these features. Chen et al. [28] extended this approach by introducing matrix factorization, and Geng et al. [29] further improved reconstruction performance by incorporating spatial constraints. However, due to the significant spectral discrepancy between RGB images and hyperspectral images, accurately representing the spectral characteristics of real-world objects under limited prior knowledge or assumptions remains a challenging task [30].

The challenge of accurately reconstructing the spectral–spatial properties of HSIs is not limited to SSR but is a recurring theme across various hyperspectral image processing tasks. For instance, in the related domain of hyperspectral image restoration, Duan et al. [31] addressed the problem of shadow removal through a multiexposure fusion framework. While targeting a different application, their work exemplifies the broader need for developing specialized techniques that can handle the complex degradation processes inherent in real-world HSI data, a challenge that also underpins the SSR problem.

Deep learning-based methods have surpassed traditional approaches due to their strong feature extraction capabilities and generalization across diverse datasets. Among these methods, CNN-based approaches are the most prevalent. The earliest deep learning method was DenseUnet proposed by Galliani et al. [32]. Subsequently, Xiong et al. [33] improved the very deep super-resolution CNN (VDSR) network [34] to develop HSCNN for SSR tasks. Later, Shi et al. [35] further enhanced this framework by constructing HSCNN-R with deep residual structures and HSCNN-D with dense connection architectures, respectively.

With the remarkable success of attention mechanisms, CNN-based models have progressively incorporated attention modules to adaptively learn more informative features, significantly enhancing network learning capabilities. For instance, Li et al. [36] proposed AWAN, which redistributes channel feature responses by integrating channel correlations to achieve more accurate reconstruction. Zhao et al. [37] designed a 4-level hierarchical regression network (HRNet), leveraging residual dense blocks for artifact removal and residual global blocks for modeling remote pixel correlation. Subsequently, Li et al. [38] introduced a deep hybrid 2D-3D CNN network with dual second-order attention (HSACS) to fully exploit sufficient spatial–spectral contextual information, achieving effective modeling of spatial–spectral dependencies. In recent work, Duan et al. [39] proposed a spectral–spatial-frequency fusion network (SSFDF), marking a first attempt to incorporate frequency-domain information into the SSR pipeline. Sun et al. [40] proposed a hybrid spectral and texture attention pyramid network, which utilizes a learnable texture feature extraction module to extract texture features from RGB images and enables comprehensive exploration of spatial–spectral correlations through spatial–spectral cross-attention.

Subsequently, the self-attention mechanism has demonstrated immense potential, and Transformer-based models have proven to be effective tools in computer vision [41]. For example, Cai et al. [42] proposed a Multi-stage Spectral-wise Transformer (MST++) to learn correlations between different spectral bands of HSI for efficient spectral reconstruction. While existing deep learning-based SSR methods integrate multiple modules and achieve visually satisfactory results, they inevitably face several challenges. First, deeper networks capable of capturing more information typically entail substantial parameters and floating-point operations. Second, advanced attention networks often operate at the expense of global receptive fields, and the dilemma of balancing computational efficiency with global modeling remains largely unresolved.

### 2.2. State Space Models

State Space Models (SSMs) [43,44], originating from control theory, were initially designed to describe and predict the dynamic evolution of systems over time [45]. Inspired by SSMs, the structured state space sequence model (S4) [43] integrates Hippo matrices with discretization operations to achieve long-range sequence modeling capabilities, demonstrating significant potential in processing sequential data. Subsequently, Fu et al. [46] bridged the efficacy gap separating SSMs from Transformers in natural language processing through their H3 architecture. Mehta et al. [47] further enhanced the representational capacity of SSMs by introducing gating mechanisms. Recently, Mamba has outperformed Transformers in natural language processing while maintaining linear scaling with input length [19].

Given Mamba’s exceptional sequence processing capabilities, recent visual tasks [21,48] have initiated preliminary attempts to adopt Mamba as foundational framework. For example, Li et al. [49] combined spatial–spectral fusion blocks with Mamba for hyperspectral image classification. Similarly, Ahmad et al. [50] used wavelet transforms combined with Mamba for hyperspectral classification. Li et al. [51] combined CNN with Vision Mamba for hyperspectral object detection. However, Mamba’s potential remains largely unexplored in the SSR domain to date.

Recent pioneering efforts have sought to adapt Mamba for SSR, yet they exhibit critical limitations in modeling the joint spectral–spatial nature of the task. Wang et al. [52] introduced gradient attention to guide Mamba for spectral reconstruction; however, their adoption of VMamba’s Cross-Scan module restricts the SSM to operate independently on single channels, failing to capture inter-spectral dependencies. In contrast, Lin et al. [53] proposed a hybrid Transformer–Mamba architecture to balance global modeling capacity and computational efficiency. Although they considered channel scanning, their “head-to-tail” serialization strategy neglects fine-grained local pixel interactions. Moreover, Lin’s method relies on a U-Net-like structure with repeated downsampling and upsampling operations, which inevitably leads to the loss of detailed spatial information. The shortcomings of these works collectively highlight a prevailing challenge: existing approaches struggle to perform continuous and lossless spectral–spatial sequence modeling. This work aims to pioneer the exploration of Mamba’s capabilities in SSR and proposes a novel perspective for the SSR task.

## 3. Method

### 3.1. Preliminaries

Structured State Space Sequence (S4) model leverages a continuous-time state space modeling framework to process discrete data, mapping the input sequence x(t)∈R to the output sequence y(t)∈R through an implicit latent state $h\left(t\right)\in R^{N}$, where *N* denotes the state space dimension. The mathematical formulation is expressed as follows:

(1) $$\begin{array}{cc} \; & h^{\prime }\left(t\right)=Ah(t)+Bx(t)\\ \; & y(t)=Ch(t)+Dx(t) \end{array}$$

The state transition matrix $A\in R^{N\times N}$ autonomously governs latent state evolution by integrating historical information to maintain system memory. $B\in R^{N\times 1}$ dynamically weights input signals to regulate their influence on latent state updates. $C\in R^{1\times N}$ transforms latent states into observable outputs, while D∈R provides direct input-to-output connections to preserve transient signal characteristics.

The continuous parameters are then discretized via the zero-order hold (ZOH) rule, integrating actual algorithms into deep learning. The definition is as follows:

(2) $$\begin{array}{cc} \; & \bar{A}=\exp(\Delta A)\\ \; & \bar{B}={\left(\Delta A\right)}^{-1}\left(\exp(A)-I\right)\cdot \Delta B \end{array}$$

where Δ represents the learnable time scale parameter for converting continuous parameters A into discrete parameters $\bar{A}$ and $\bar{B}$. Then Equation (1) can be rewritten as follows:

(3) $$\begin{array}{cc} \; & h_{t}=\bar{A}h_{t-1}+\bar{B}x_{t}\\ \; & y_{t}=Ch_{t}+Dx_{t} \end{array}$$

In addition, Equation (3) can also be transformed into convolution form:

(4) $$\begin{array}{cc} \; & \bar{K}\overset{\Delta }{=}\left(C\bar{B},C\bar{AB},\cdots ,C{\bar{A}}^{L-1}\bar{B}\right)\\ \; & y=x*\bar{K} \end{array}$$

Among them, $\bar{K}\in R^{L}$ is the structured convolution kernel, *L* is the length of the input sequence, and ∗ represents the convolution operation.

The S4 framework achieves enhanced computational efficiency through dynamic parameter adaptation, a capability further refined in its advanced iteration S6 (Mamba). By rendering parameters B, C and Δ input-dependent, Mamba enables context-aware processing tailored to varying input characteristics.

### 3.2. Overall Architecture

**Figure 1:**
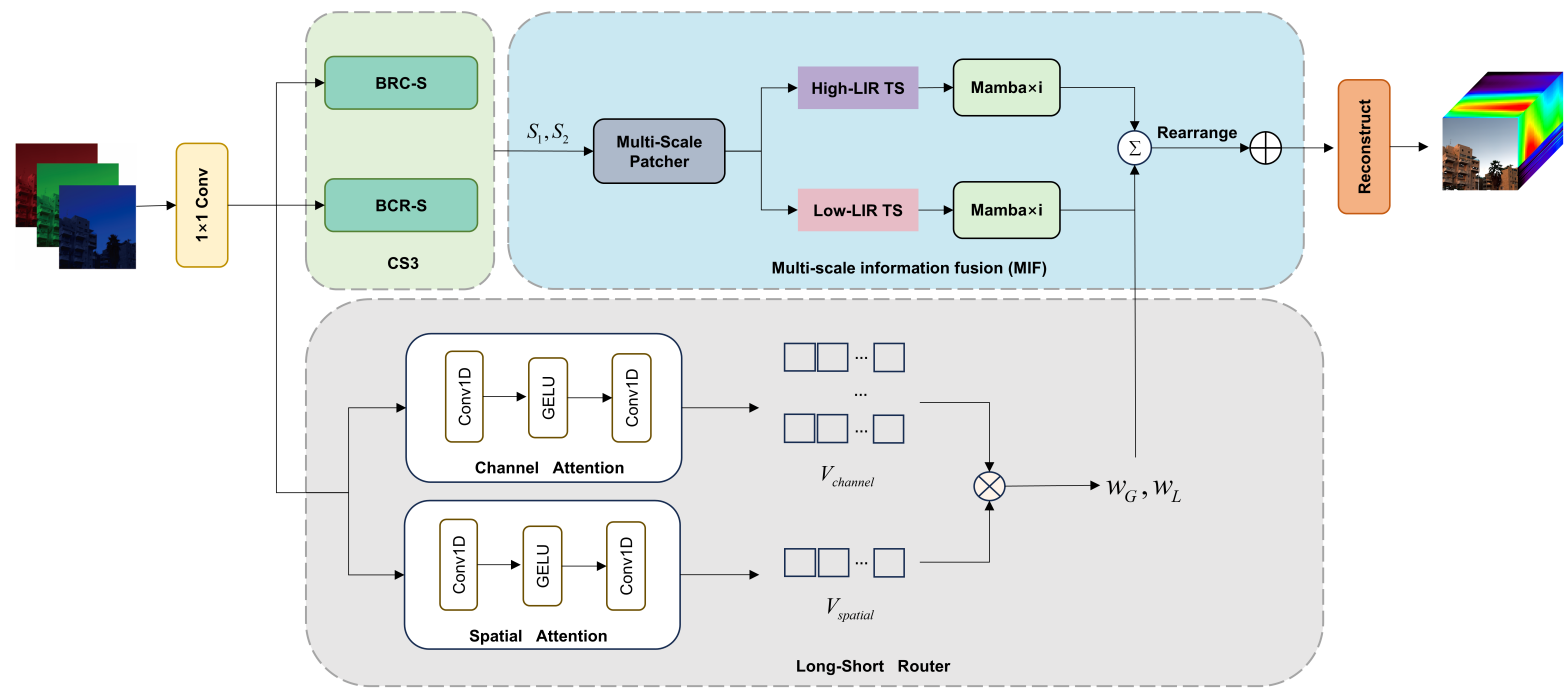
The overall network architecture of the proposed Multi-scale Spectral–Spatial Sequence Learning Mamba (MSS-Mamba).

As illustrated in Figure 1, MSS-Mamba architecture comprises four core components: shallow feature extraction, continuous spectral–spatial scan (CS3), multi-scale information fusion (MIF) and high-quality reconstruction. The network processes a single RGB image $I_{R}\in R^{3\times H\times W}$ as input. During shallow feature extraction, 1×1 2D convolution initially extract low-level feature maps $F_{S}\in R^{C\times H\times W}$, where C×H×W represents channel depth, height and width.

(5) $$\begin{array}{c} F_{S}=\mathrm{Conv}2D\left(I_{R}\right) \end{array}$$

The shallow features $F_{S}$ are subsequently fed into the CS3 module to generate sequential representations $S_{n}$, which encode rich spatial–spectral correlations for robust multi-scale feature extraction. These sequences $S_{n}$ are then processed by the MIF module to learn high-level discriminative features $F_{D}$. The entire process can be described as follows:

(6) $$\begin{array}{cc} \; & S_{1}=\mathrm{BRC}\text{-}S\left(F_{S}\right)\\ \; & S_{2}=\mathrm{BCR}\text{-}S\left(F_{S}\right)\\ \; & F_{D}=\mathrm{MIF}\left(S_{1}\right)+\mathrm{MIF}\left(S_{2}\right) \end{array}$$

The CS3 is divided into two scanning modes: BRC-S and BCR-S. Finally, the high-quality reconstruction stage synthesizes $F_{D}$ into high-fidelity hyperspectral images $I_{H}$:

(7) $$\begin{array}{c} I_{H}=\mathrm{Reconstruction}\left(F_{S}+F_{D}\right) \end{array}$$

### 3.3. Continuous Spectral–Spatial Scan

Original Mamba demonstrates remarkable advantages in long-sequence modeling tasks, particularly in global receptive field establishment and long-range dependency learning. However, its application to image processing necessitates specialized scanning strategies to transform multidimensional data into 1D sequences. Existing vision-oriented scanning methods primarily focus on spatial dimensions, which exhibit critical limitations in adaptability when extended to 3D hyperspectral data characterized by intricate spatial–spectral interdependencies. To address this, we propose CS3, a novel technique that generates sequences rich in spatial–spectral information to accommodate the complexity of 3D image data while preserving cross-dimensional correlations.

**Figure 2:**
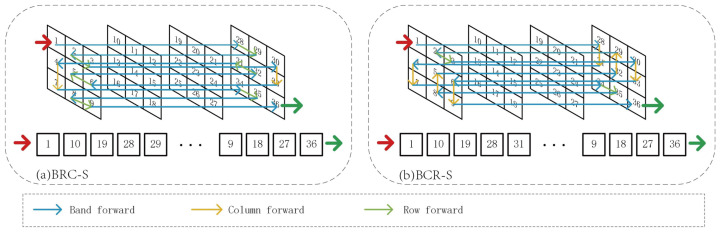
Continuous spectral–spatial scan. The (**a**) band–row–column scan strategy; the (**b**) band–column–row scan strategy.

As illustrated in Figure 2, the CS3 strategy unfolds 3D feature maps in a pixel-wise manner, where each pixel has dependency relationships with surrounding pixels across rows, columns and bands. Taking BRC-S as an example, the input 3D feature map $F_{S}\in R^{C\times H\times W}$ undergoes dimensional rearrangement to $F_{S_{1}}\in R^{H\times W\times C}$, which is then scanned into sequence $S_{1}\in R^{H\times L}$ with L=W×C. This process sequentially scans each column following band and row orders. Specifically, BRC-S first scans band-row planes before proceeding to column traversal, as visualized in Figure 2a. The generated sequences preserve inter-pixel dependencies through tail-to-tail or head-to-head concatenation, where adjacent elements in the sequence maintain spatial–spectral correlations.

Mamba’s unidirectional recurrent processing propagates dependencies solely from preceding tokens, potentially isolating spatially proximate pixels in distant sequence positions and causing local information degradation. To mitigate this, as visualized in Figure 2b, BRC-S introduces band-column-row scanning to create complementary sequences that enhance long-range modeling diversity. Collectively, CS3 innovatively encodes cross-dimensional relationships into sequences through intelligent dimensional permutations, effectively exploiting spatial–spectral synergies.

### 3.4. Multi-Scale Information Fusion

While Transformer-based multi-scale learning frameworks improve efficiency through image patch partitioning, they inherently suffer from two critical limitations: inadequate interaction between global and local contextual features [42], and persistent incompatibility between global receptive fields and computational efficiency. Addressing these challenges, our Multi-scale Information Fusion (MIF) block introduces a sequence tokenization approach that performs multi-scale slicing operations prior to feeding scanned sequences into Mamba. Inspired by temporal sequence modeling principles in Mamba, this strategy processes hierarchical visual patterns through adaptive window slicing while preserving sequence continuity. The proposed method significantly enhances cross-scale feature interactions by jointly optimizing multi-granularity pattern extraction and sequence dependency propagation, thereby achieving synergistic integration of global contextual awareness and local detail preservation within an efficient computational paradigm.

**Figure 3:**
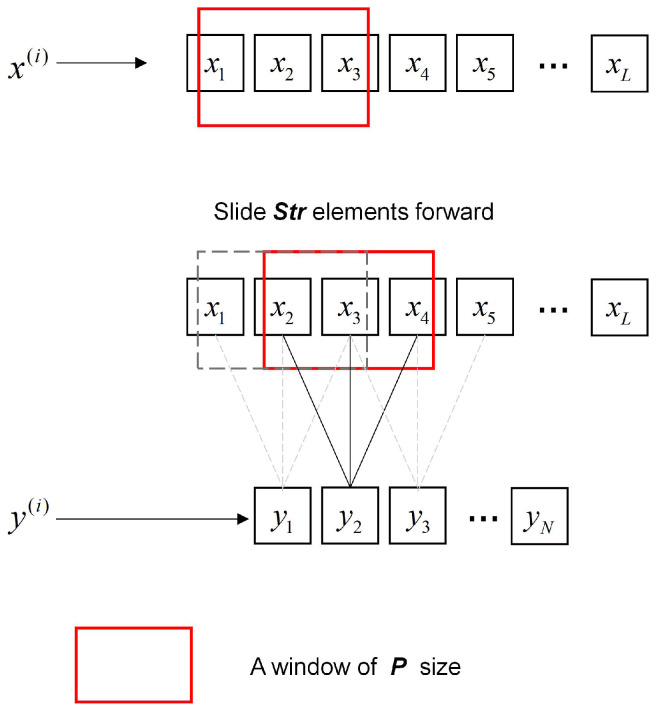
Sliding window slicing in Multi-Scale Patch.

Multi-Scale Patch: As shown in Figure 1, the sequences generated by CS3 are first fed into a multi-scale patch generator. This module produces multi-scale sequences by configuring varying parameters for sequence tokenization. Specifically, larger patches retain more spatial pixels, enhancing the model’s understanding of structural trends and holistic contextual relationships to facilitate global spatial modeling. Conversely, smaller patches encompass more spectral channel pixels, strengthening the model’s ability to learn variation trends and correlations in adjacent spectral bands for local spectral modeling. To ensure effective spectral–spatial correlation learning, the patch size is set to exceed the number of feature channels. The specific form is shown in Figure 3. Formally, consider the sequence $S_{1}=\left\{x^{\left(1\right)},x^{\left(2\right)},\ldots ,x^{\left(H\right)}\right\}\in R^{L\times H}$ generated by BRC-S, where each column vector $x^{\left(i\right)}\in R^{L\times 1}$ represents a spectral–spatial feature stream. The sequence tokenization process applies sliding window slicing with patch length *P* and stride Str, generating N=⌊(L−P)/Str⌋+1 patches per column. This operation transforms each $x^{\left(i\right)}$ into a patch matrix $y^{\left(i\right)}\in R^{N\times P}$, effectively expanding the original 2D sequence $S_{1}$ into a 3D tensor $S_{1}^{\prime }\in R^{H\times N\times P}$. Here, *N* becomes the new sequence dimension encoding multi-scale contextual hierarchies, while *P* serves as the variable dimension capturing localized pattern characteristics.

Local Information Richness (LIR): To measure the richness of local spectral information in a sequence, we propose a new metric called LIR. The specific calculation method is as follows:

(8) $$\begin{array}{c} \mathrm{LIR}=N\left(P-Str\right)=\left(\left\lfloor \frac{L-P}{Str}\right\rfloor +1\right)\left(P-Str\right)\approx \frac{\sqrt{P}}{Str} \end{array}$$

The LIR metric is intrinsically governed by two critical parameters, *P* and Str, which jointly determine its computational characteristics. The LIR value is positively correlated with local spectral information density — higher LIR values indicate richer localized patterns within sequences. Notably, patch dimensions differentially modulate receptive fields: larger *P* expands spatial–spectral coverage, enabling efficient global spatial modeling with reduced computational overhead while maintaining stride sizes. Conversely, smaller *P* prioritizes localized spectral information capture through decreased Str, which increases inter-patch overlaps to enhance fine-grained feature extraction. This parametric flexibility allows LIR to adaptively balance global contextual learning and local discriminative analysis.

The entire multi-scale patch process can be described by the following formula:

(9) $$\begin{array}{c} S_{L},S_{G}=\mathrm{MP}\left(S_{n},P_{L},Str_{L}\right),\mathrm{MP}\left(S_{n},P_{G},Str_{G}\right) \end{array}$$

$S_{L}$ and $S_{G}$ represent the generated high and low LIR sequences, respectively. MP is the multi-scale patch generator. $P_{L},Str_{L},P_{G}$ and $Str_{G}$ represent different slice sizes, with $P_{G}>P_{L}$ and $Str_{G}>Str_{L}$.

Long-Short Router: The proposed Long-Short Router module dynamically allocates computational resources by learning adaptive, input-dependent weights to integrate global and local representations. As shown in Figure 1, unlike traditional Mixture-of-Experts (MoE) routers that perform discrete path selection, our router learns the continuous relative contributions of two complementary pathways through a dual-attention mechanism followed by adaptive interpolation.

Formally, given the input feature map $F_{S}\in R^{C\times H\times W}$ from the shallow extraction module, it is first reshaped into a token sequence $X=\mathrm{reshape}\left(F_{S}\right)\in R^{C\times M}$ where M=H×W indexes the spatial locations. This sequence X is then processed by the router to generate pathway weights. The router leverages parallel channel-wise and spatial-wise attention branches to capture both semantic and structural information, generating a comprehensive representation:

(10) $$\begin{array}{cc} \; & V_{\mathrm{channel}}=\phi_{\theta_{1}}\left(S_{1}\right),\\ \; & \;V_{\mathrm{spatial}}=\psi_{\theta_{2}}\left(S_{1}\right) \end{array}$$

where $\phi_{\theta_{1}}$ denotes a pointwise convolution (1×1) for channel attention, and $\psi_{\theta_{2}}$ denotes a depthwise convolution (3×3) for spatial attention. These two attentions are combined via element-wise multiplication to form a joint feature representation $V=V_{\mathrm{channel}}⊙V_{\mathrm{spatial}}$.

Subsequently, the combined representation is aggregated via global average pooling and projected to a 2-dimensional weight vector:

(11) $$\begin{array}{cc} \; & w=\mathrm{AdaptiveAvgPool}1d(V),\\ \; & \;\left[w_{L},w_{G}\right]=\mathrm{softmax}\left(f_{\theta_{3}}\left(w\right)\right) \end{array}$$

where $f_{\theta_{3}}$ is a linear projection layer. The final output weights $w_{L},w_{G}\in \left(0,1\right)$ are broadcasted and applied to modulate the contributions of the local and global pathways, respectively. This design enables instance-specific resource allocation without the need for discrete routing or expert selection.

Then, the multi-scale sequences generated by Equation (9) are input into Mamba for feature learning. The feature maps obtained are weighted and summed by the weights generated from Equation (11). Finally, the depth feature map is restored to the input format via restore. The specific formulas are as follows:

(12) $$\begin{array}{c} F_{M}=\mathrm{Restore}\left(w_{L}\cdot f_{\mathrm{Mamba}}^{\left(i\right)}\left(S_{L}\right)+w_{G}\cdot f_{\mathrm{Mamba}}^{\left(i\right)}\left(S_{G}\right)\right) \end{array}$$

where $F_{M}\in R^{C\times H\times W}$ is the output depth feature map, and $f_{\mathrm{Mamba}}^{\left(i\right)}$ represents the *i*-th Mamba layer in the hierarchical stack. Finally, the feature maps from different sequences are element-wise summed via Equation (6), and a high-quality image is reconstructed via Equation (7).

## 4. Experiments

### 4.1. Dataset and Evaluation Metrics

The proposed MSS-Mamba is evaluated on three widely recognized hyperspectral reconstruction benchmarks: the ARAD_1k dataset from NTIRE 2022 competition [54], the CAVE dataset [55] and the IEEE *grss_dfc_2018* dataset.

The ARAD1K dataset consists of 1000 aligned RGB and hyperspectral image pairs with a spatial resolution of 512 × 482 pixels. This dataset is designed specifically for large-scale spectral recovery tasks and covers most application scenarios. Following the official competition protocol, 900 image pairs are allocated for training and 50 for validation to rigorously assess generalization performance on unseen data.

The CAVE dataset serves as a standard benchmark for few-shot hyperspectral imaging research. It contains 32 high-resolution (512 × 512 pixels) hyperspectral cubes captured under laboratory conditions, covering diverse real-world materials such as fabrics, paints and organic objects. We adopt a standardized split with 22 images for training and 10 for testing, simulating scenarios with limited training samples.

IEEE *grss_dfc_2018* dataset was collected by the National Center for Airborne Laser Mapping (NCALM) from Houston University [56]. It comprises a hyperspectral image with a spatial resolution of 4172 × 1202 pixels and 48 spectral bands covering the wavelength range of 380–1050 nm. Following the configuration in [57], bands 23, 12 and 5 were selected to synthesize the RGB image as input. The dataset was cropped into 27 paired 512 × 512 patches, with three non-overlapping patches reserved for the testing set.

To quantitatively evaluate the performance of MSS-Mamba, five widely adopted metrics were employed: the Mean Relative Absolute Error (MRAE) and Root Mean Square Error (RMSE) to quantify spectral reconstruction accuracy, Peak Signal-to-Noise Ratio (PSNR) to assess spatial fidelity, Spectral Angle Mapper (SAM) to measure spectral angular deviations and Structural Similarity Index (SSIM) to evaluate structural preservation. Specifically, lower values of MRAE, RMSE and SAM indicate reduced spectral distortions, while higher PSNR and SSIM values reflect superior spatial consistency and perceptual quality. These metrics collectively provide a rigorous multi-dimensional assessment of hyperspectral reconstruction performance, spanning spectral accuracy, spatial fidelity and structural integrity, thereby comprehensively validating the model’s effectiveness in spectral recovery tasks.

### 4.2. Parameter Setting

For the proposed MSS-Mamba network, we set a low-LIR (LIR=0.13, P=256, Str=128) for sliding window slicing as the global information sequence and a high-LIR (LIR=0.18,P=128, Str=64) as the local information sequence. The number of Mamba layers is set to 5. The original samples are cropped to produce 32 × 32 RGB and HSI pairs, with an overlap of 8 pixels, the initial feature dimension is set to 128. The ARAD1K dataset was trained for 100 epochs with 5000 iterations per epoch, while the CAVE and Houston datasets were trained for 50 epochs due to their smaller data volumes. Optimization used Adam with an initial learning rate of $1\times 10^{-4}$, dynamically annealed to $1\times 10^{-6}$ via cosine scheduling, gradually reducing parameter update magnitudes as the model approaches convergence to achieve coarse-to-fine optimization.

### 4.3. Comparison with Other Methods

The proposed method is rigorously compared with eight Cutting-edge approaches on each dataset: DenseUnet [32], HSCNN+ [35], sRCNN [58], HRNet [37], GDNet [59], SSDCN [60], GMSR [52] and SSRMamba [61]. All comparative methods adhere to standardized dataset splits and utilize publicly released implementations with pre-trained models to ensure reproducibility. This comprehensive benchmarking framework validates the method’s robustness across diverse hyperspectral reconstruction scenarios while maintaining strict experimental parity in training configurations, evaluation criteria and computational environments.

#### 4.3.1. Quantitative Results

Table 1, Table 2 and Table 3 presents the quantitative results on three datasets. It is evident from the table that our proposed MSS-Mamba consistently outperforms other methods across most of the evaluation metrics. On the ARAD1K dataset, MSS-Mamba demonstrates significant advantages by achieving optimal performance in MRAE, PSNR and SSIM. Our approach reduces spectral distortion with a 3.25% improvement in MRAE over the nearest competitor (SSDCN) and enhances reconstruction fidelity with a 1.73% PSNR gain against GMSR. However, it ranks slightly lower on SAM and RMSE. We attribute this to a inherent trade-off in our model’s design: the MSS-Mamba prioritizes global spectral–spatial consistency and perceptual quality (reflected in PSNR/SSIM), which may slightly relax the constraint on per-pixel spectral angle accuracy (SAM) in highly heterogeneous regions. The complex real-world scenes in ARAD1K make this trade-off more apparent. Nevertheless, our method maintains highly competitive performance across all metrics while providing superior overall reconstruction fidelity.

MSS-Mamba leads all metrics on both the CAVE and Houston datasets. On CAVE, it shows major gains in PSNR and SAM, proving effective in spatial–spectral modeling. On Houston, it reduces MRAE by 7.99% over DenseUnet and RMSE by 5.32% over GDNet, demonstrating strong generalization. The method simultaneously improves spectral accuracy and spatial quality, setting a new state-of-the-art for robust hyperspectral imaging across diverse data environments.

#### 4.3.2. Visual Results

**Figure 4:**
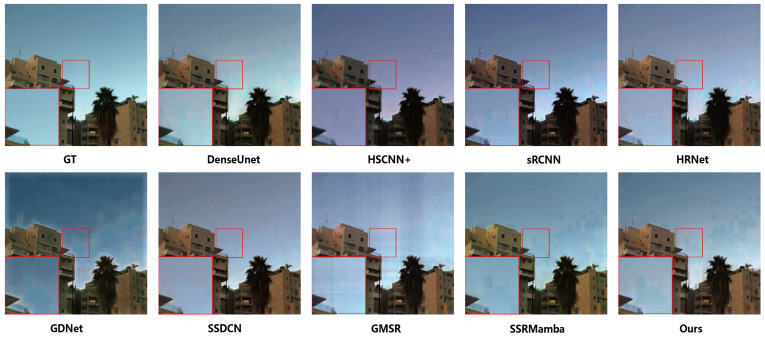
True-color synthesis images (bands 27, 17 and 10) of a sample from the ARAD1K dataset, comparing the results of different methods.

**Figure 5:**
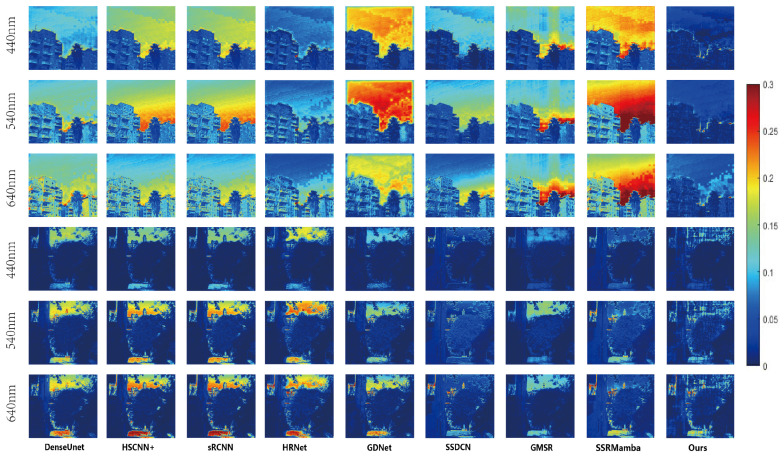
Comparative visualization across three spectral bands for architectural and vegetation samples from the ARAD1K dataset. Per-pixel reconstruction error is derived from MRAE values between super-resolution and ground-truth spectral vectors. MRAE heat maps are display-scaled for optimal visualization.

For the visual inspection of HSI reconstruction outcomes, we designed different visualization comparison graphs.On the ARAD1K dataset, Figure 4 and Figure 5 present true-color composites (bands 27, 17 and 10) along with the corresponding MRAE error maps. As shown in Figure 4, while the true-color composites show that all methods produce generally plausible results, close examination reveals that GDNet exhibits noticeable global color distortion, indicative of a fundamental spectral miscalibration. HSCNN+ and HRNet show more localized color artifacts on building surfaces, suggesting difficulties with material-specific spectral reconstruction.

The MRAE maps in Figure 5, however, are far more discriminative. Two key observations can be made: (1) Spectral Consistency in Homogeneous Regions: The sky region exhibits high error (intense red) for most methods. This indicates a widespread failure to model subtle spectral variations in low-texture areas. Our method shows a marked reduction in error here. This can be directly attributed to the CS3 module’s continuous scanning strategy, which traverses the spatial and spectral dimensions simultaneously. Unlike patch-based methods that may break the continuity of the sky region, our approach maintains a global contextual understanding, allowing it to model these subtle, large-scale spectral variations more effectively. (2) Spatial–Spectral Complexity in Vegetation: In contrast to DenseUnet and HSCNN+, which produce spatially coherent errors indicating a fundamental misrepresentation of the vegetation’s structure, our method exhibits a more diffuse and lower-magnitude error pattern. This suggests that the MIF block successfully fuses multi-scale features to better represent the complex interplay of texture and spectral signature inherent in natural scenes, thereby avoiding such structured artifacts.

**Figure 6:**
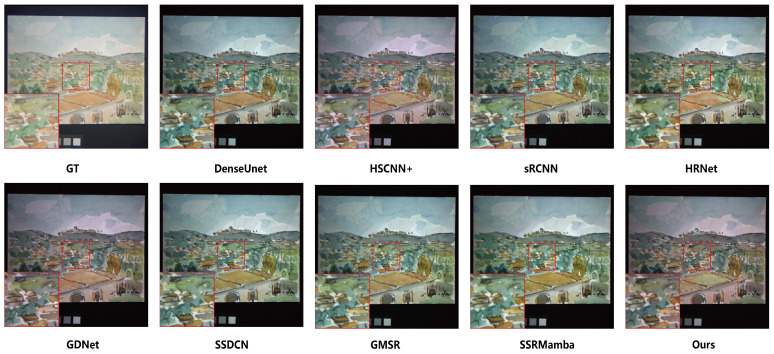
True-color synthesis images (bands 27, 17 and 10) of a sample from the CAVE dataset, comparing the results of different methods.

**Figure 7:**
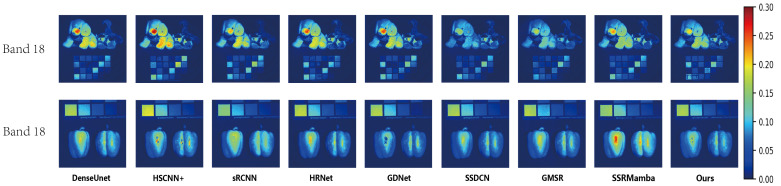
Visual reconstruction results from two different images from the CAVE dataset, each taken from band 18. Per-pixel reconstruction error is derived from MRAE values between super-resolution and ground-truth spectral vectors. MRAE heat maps are display-scaled for optimal visualization.

The reconstruction results on the CAVE dataset are presented in Figure 6 and Figure 7. Figure 6 displays the true-color composite images (bands 27, 17 and 10). While the outputs from all compared methods are visually plausible and exhibit high perceptual quality at a glance, making fine-grained distinctions challenging based on RGB visualization alone, the corresponding MRAE error maps in Figure 7 provide a more objective and discriminative assessment. The minimal visual discrepancy in color composites underscores the challenging nature of this benchmark and necessitates a quantitative evaluation to uncover perceptually subtle yet critical differences in spectral–spatial fidelity. As revealed in Figure 7, several deep learning-based methods exhibit pronounced errors in specific regions, such as the teddy bear’s nose and the surface of the chili pepper. In comparison, SSDCN, GMSR and our method achieve relatively lower error levels overall. Notably, our approach demonstrates superior performance in detail preservation and spectral continuity—particularly evident in these structurally and spectrally complex regions—affirming the efficacy of the proposed joint spectral–spatial scanning strategy.

**Figure 8:**
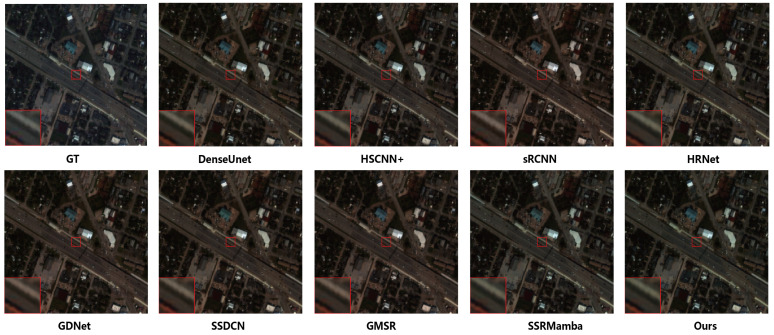
True-color synthesis images (bands 23, 12 and 5) of a sample from the IEEE *grss_dfc_2018* dataset, comparing the results of different methods.

**Figure 9:**
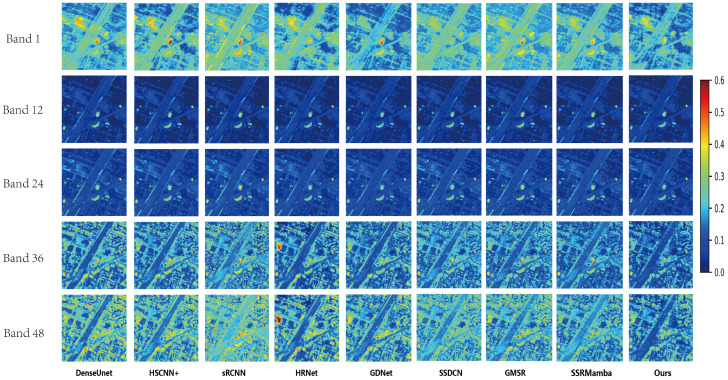
Absolute differences between the reconstructed images and the ground truth at bands 1, 12, 24, 36 and 48 on the IEEE *grss_dfc_2018* dataset. The scale values on the color bar on the right side represent the absolute difference divided by the maximum possible value in the reconstructed HSI.

Figure 8 illustrates the true-color composites (bands 23, 12 and 5) from the IEEE grss_dfc_2018 dataset. Figure 9 further provides absolute error maps between the reconstructed results of each method and the ground-truth references. Overall, all methods perform satisfactorily in reconstructing mid-band wavelengths, whereas their adaptability diminishes toward both lower and higher bands, underscoring the challenging nature of the Houston dataset. Certain methods, such as HRNet, exhibit significant errors in particular regions (e.g., buildings in higher bands), indicating limited generalization capability and regional adaptability. In contrast, the proposed MSS-Mamba method maintains consistently lower error levels across various bands and regions, further confirming its ability to reconstruct both global structures and fine local details with high fidelity.

**Figure 10:**
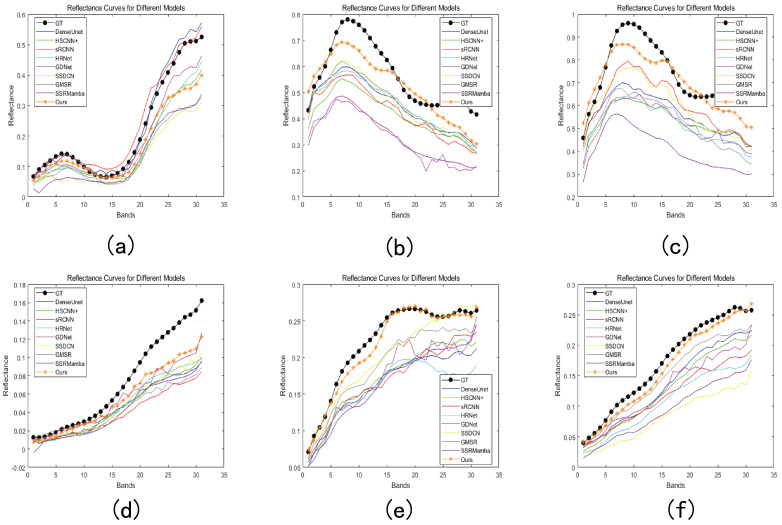
Spectral curves on six objects generated by different methods. (**a**) Red flowers. (**b**) Building. (**c**) Trees. (**d**) Pottery jar. (**e**) Graffiti. (**f**) Soil wall.

To investigate MSS-Mamba’s spectral reconstruction capability across diverse surfaces, we compared spectral reflectance curves across 31 bands between ground-truth data and reconstructed images from various methods. As shown in Figure 10, spectral reflectance profiles from six representative locations demonstrate that MSS-Mamba achieves the closest alignment with ground-truth measurements-particularly for challenging surfaces like pottery jars (Figure 10d) and Soil wall (Figure 10f). These results validate MSS-Mamba’s superior performance in reconstructing complex spectral signatures compared to alternative approaches. It is worth noting that the reconstruction fidelity can vary depending on the material properties and spectral characteristics of the region. While our method demonstrates robust performance in most areas, certain materials with specific spectral signatures in high-frequency bands present a valuable challenge for future work.

### 4.4. Ablation Analysis

#### 4.4.1. Multi-Scale Information Learning

To investigate the impact of sequence modeling strategies on multi-scale feature learning, we conducted three ablation studies on the ARAD_1K dataset. First, to validate the effectiveness of global and local sequence modeling, experiments were performed by retaining only the global or local information learning modules. Second, the contribution of the adaptive weighting fusion module was analyzed by disabling this component.

As shown in Table 4, the full model achieves optimal performance. Among them, in the global mode, MSS-Mamba outperforms the local mode in terms of PSNR, but SAM is lower then local mode. This indicates that the global mode is more effective in preserving the overall structure of the image, while the local mode can more accurately estimate the relative changes in the spectrum.Integrating global and local modules without adaptive fusion yields intermediate results, highlighting their complementary roles. Enabling adaptive fusion further refines performance, there has been a significant improvement in each indicator. These results validate the necessity of adaptive weighting for balancing multi-scale spectral–spatial features, ultimately achieving state-of-the-art reconstruction fidelity.

#### 4.4.2. The Effectiveness of CS3

To investigate the effects of CS3 combined with foundation model(Base) on the model, we designed four sets of experiments: using the foundation model of the original sequence instead of CS3 (Non-CS3), Base combined with BRC-S, Base combined with BCR-S and Base combined with CS3. The experiments were conducted on the ARAD1K dataset.

As summarized in Table 5, The combined implementation of BRC-S and BCR-S with the Base model achieves optimal performance, underscoring the effectiveness of our multi-directional scanning strategy. Although the single-sequence model—particularly Base+BCR-S—exhibits a marginal advantage in SAM metrics, highlighting the strength of CS3 scanning in reconstructing and exploring spectral information, it falls short in capturing fine spatial details, as reflected in lower PSNR and SSIM scores. In contrast, non-CS3 configurations underperform across all metrics. These results emphasize the limitations of conventional scanning sequences in spatial–spectral feature extraction: their failure to preserve continuous information flow results in the gradual degradation of spectral–spatial features throughout processing.

#### 4.4.3. Different Number of Mamba Layers

To validate the efficacy of stacking Mamba layers in MSS-Mamba, we conducted ablation studies with varying layer depths (1–6) on the ARAD1K dataset. As detailed in Table 6, increasing Mamba layers raises parameter count by approximately 0.88 M per layer. Configurations with one or two layers exhibit limited representational capacity, resulting in compromised recovery of complex spectral–spatial features. The 3-layer configuration demonstrates strong and competitive performance, particularly in spectral accuracy: it achieves the best MRAE and a very low SAM, indicating excellent spectral fidelity with minimal distortion. The fact that SAM does not improve substantially with deeper architectures suggests that the 3-layer model already captures essential spectral characteristics effectively. With only 3.12 M parameters, it offers an attractive balance between efficiency and reconstruction quality, making it highly suitable for applications that prioritize spectral precision and computational economy.

The 5-layer model, while requiring 1.76M more parameters, delivers superior overall reconstruction quality, excelling in perceptually critical metrics including PSNR and SSIM. It also maintains strong all-around performance without significant degradation in any metric, indicating better generalization across diverse spectral and spatial features. While the 6-layer model achieves marginally higher PSNR and lower RMSE, the performance gain over the 5-layer model is minimal compared to the additional parameter cost, suggesting diminishing returns beyond five layers.Thus, the 3-layer model stands out as a compact and spectrally accurate configuration, whereas the 5-layer version provides the optimal trade-off for high-fidelity reconstruction across both spatial and spectral domains, justifying its selection as our primary architecture.

#### 4.4.4. Different Fusion Methods

To evaluate the impact of our proposed routing mechanism, we compared three fusion strategies: element-wise addition (Add), a simple gating mechanism (Gate) and an attention-enhanced gating mechanism (Gate+Att). As summarized in Table 7, the attention-based gating approach achieves the best overall performance, attaining optimal values in MRAE, RMSE, PSNR and SSIM. Although the plain gating mechanism yields a slightly better SAM, the minor degradation in our method is likely due to the attention mechanism prioritizing broader spatial–spectral contextual integration over extreme angular accuracy. In contrast, simple addition fails to adaptively weight features, resulting in consistently inferior performance across most metrics and confirming its limited capacity for effective spatial–spectral fusion.

## 5. Conclusions

This study proposes a multi-scale spectral–spatial sequence learning SSR network MSS-Mamba, which integrates continuous spectral–spatial scanning mechanism with adaptive multi-scale feature fusion strategy. Specifically, MSS-Mamba achieves collaborative optimization of global structural consistency learning and local spectral sensitivity through a dual path architecture, combined with complementary scanning modes of BRC-S and BCR-S, significantly enhancing the diversity expression of spatial–spectral features. The adaptive weight fusion module further dynamically balances the contribution of multi-scale information, overcoming the limitations of static fusion strategies. In future work, we plan to further refine the model architecture to enhance its performance and applicability. Several specific directions are envisioned:

First, while the proposed CS3 scanning strategy effectively captures continuous spectral–spatial dependencies—addressing a key limitation of current scanning approaches—it may still disrupt certain local spatial relationships. To mitigate this, we will explore more rigorous scanning mechanisms and investigate the integration of more effective positional encoding techniques to better preserve structural integrity.

Second, the sliding-window slicing strategy introduced in this work reduces the reliance on down-sampling for multi-scale feature learning in visual Mamba. Building on this idea, we aim to design more comprehensive and adaptive slicing schemes to capture richer contextual information across scales.

Finally, we will focus on developing lightweight and efficient variants of the model that demand minimal computational resources, thereby improving the practicality and deployment potential of spectral super-resolution methods in real-world applications.

## Figures and Tables

**Table 1:** Performance of different methods on the ARAD1K dataset.

Model	MRAE (↓)	RMSE (↓)	PSNR (↑)	SAM (↓)	SSIM (↑)
DenseUnet	0.4567	0.0634	34.9083	6.5300	0.9652
HSCNN+	0.4371	0.0638	34.9083	6.6335	0.9695
sRCNN	0.4592	0.0663	34.3201	7.6121	0.9679
HRNet	0.4134	0.0608	35.1461	6.6606	0.9756
GDNet	0.4250	0.0590	34.9685	8.5381	0.9694
SSDCN	0.3595	0.0558	35.7012	7.9082	0.9509
GMSR	0.3821	0.0566	35.7286	7.8836	0.9739
SSRMamba	0.3724	0.0632	34.6764	6.8082	0.9634
Ours	0.3478	0.0569	36.3473	6.7223	0.9790

**Table 2:** Performance of different methods on the CAVE dataset.

Model	MRAE (↑)	RMSE (↑)	PSNR (↑)	SAM (↑)	SSIM (↑)
DenseUnet	0.2217	0.0502	42.2622	9.7953	0.9592
HSCNN+	0.2150	0.0539	42.2452	10.1816	0.9565
sRCNN	0.2232	0.0521	42.1584	10.7899	0.9588
HRNet	0.2043	0.0515	42.8256	9.7335	0.9587
GDNet	0.2326	0.0517	42.1163	10.0515	0.9578
SSDCN	0.2202	0.0510	42.4011	9.9417	0.9593
GMSR	0.2218	0.0495	42.2899	9.7561	0.9586
SSRMamba	0.2191	0.0494	42.1625	10.4200	0.9545
Ours	0.2042	0.0492	42.9930	9.6751	0.9616

**Table 3:** Performance of different methods on the Houston dataset.

Model	MRAE (↑)	RMSE (↑)	PSNR (↑)	SAM (↑)	SSIM (↑)
DenseUnet	0.1990	0.0564	36.6269	7.8734	0.9884
HSCNN+	0.2018	0.0591	36.3841	8.0927	0.9874
sRCNN	0.2478	0.0714	34.8570	9.6955	0.9822
HRNet	0.2087	0.0593	36.7932	7.9343	0.9899
GDNet	0.2133	0.0564	36.1574	8.2554	0.9892
SSDCN	0.2276	0.0589	36.0359	8.4328	0.9890
GMSR	0.2269	0.0578	36.0322	8.2670	0.9902
SSRMamba	0.2064	0.0607	35.5673	8.6418	0.9882
Ours	0.1831	0.0534	37.0063	7.7882	0.9924

**Table 4:** Ablation experiments on the effect of introduced components on ARAD1K dataset.

Global	Local	Router	MRAE (↓)	RMSE (↓)	PSNR (↑)	SAM (↓)	SSIM (↑)
✓	**✗**	**✗**	0.3696	0.0648	35.2076	7.8570	0.9649
**✗**	✓	**✗**	0.3934	0.0612	34.5004	6.6077	0.9796
✓	✓	**✗**	0.3673	0.0591	35.5395	6.7615	0.9729
✓	✓	✓	0.3478	0.0569	36.3473	6.7223	0.9790

**Table 5:** Ablation experiments for different scan modes on ARAD1K dataset.

Scan Mode	MRAE (↓)	RMSE (↓)	PSNR (↑)	SAM (↓)	SSIM (↑)
Non-CS3	0.3696	0.0648	35.2076	7.8570	0.9649
Base+BRC-S	0.3415	0.0604	35.8563	6.4660	0.9734
Base+BCR-S	0.3380	0.0576	35.5562	6.0146	0.9664
Base+CS3	0.3478	0.0569	36.3473	6.7223	0.9790

**Table 6:** Ablation experiments of Mamba layer on ARAD1K dataset.

Mamba Layer	Params (M)	MRAE (↓)	RMSE (↓)	PSNR (↑)	SAM (↓)	SSIM (↑)
1	1.37	0.3554	0.0571	34.9126	7.4812	0.9638
2	2.25	0.3462	0.0609	35.3576	7.0128	0.9679
3	3.12	0.3419	0.0556	35.5242	6.8126	0.9703
4	4.01	0.3965	0.0585	35.6379	6.7478	0.9836
5	4.88	0.3478	0.0569	36.3473	6.7223	0.9790
6	5.75	0.3420	0.0522	36.6795	6.6259	0.9763

**Table 7:** Ablation experiments for different fusion methods on ARAD1K dataset.

Fusion Method	MRAE (↓)	RMSE (↓)	PSNR (↑)	SAM (↓)	SSIM (↑)
Add	0.3517	0.0607	35.6741	7.2536	0.9671
Gate	0.3773	0.0595	35.6517	6.7073	0.9672
Gate+Att	0.3478	0.0569	36.3473	6.7223	0.9790

## Data Availability

Data will be made available on request.
